# Grand Challenges in Biomaterials

**DOI:** 10.3389/fbioe.2014.00043

**Published:** 2014-10-17

**Authors:** Hasan Uludağ

**Affiliations:** ^1^University of Alberta, Edmonton, AB, Canada

**Keywords:** biomaterials, regeneration, molecular design, biomimicry, biocompatibility

The biomaterials field constitutes a transdisciplinary activity in which synthetic or naturally derived materials are utilized for a variety of desired outcomes in living systems. Biomaterials may act as physical supports at times, providing a space in which biological systems manifest their inherent characteristics. More commonly, biomaterials actively participate in the performance of a biological system, supporting or even inducing desirable traits that would have not been possible without the biomaterial. While biomaterials have become an integral part of several industrial processes and products, it is their medical use in the body that fascinated and stimulated our imagination. From simple entrapment of enzymes (Chang, 1964), we witnessed the creation of “bioartificial” organs combining cells and materials (Chick et al., 1977), followed by enzyme-sensitive biomaterials by design (Ulbrich et al., 1980), and engineered materials that matched the physical (Urry et al., 1981) and cellular requirements (Massia and Hubbell, 1991) of injured tissues. We learned to utilize biomaterials to control the release of small molecules such as drugs (Schwartz et al., 1968; Goodman and Banker, 1970) and especially macromolecules such as proteins (Langer and Folkman, 1976), which stimulated us to explore unique therapies. The potential of biomaterials is manifested in diverse areas and every organ in our bodies have benefited from them in one form or another. To fulfill such a broad translational promise, one needs to rely on sound scientific and engineering practice and should be exceptionally vigilant in fabricating and assessing the constructed systems. Identifying a few unifying (fundamental) principles for developing functional biomaterials is difficult when the biomaterials are intended for so many different uses, whether to stimulate our body’s surveillance system to fight malignant cells or infections, to restore the physical barrier in skin upon losing its integrity, or to act as a blood conduit in tiny channels. I believe that the challenges facing functional biomaterials need to be addressed in the context of intended applications. However, I would like to propose that one should be mindful of two broad challenges irrespective of the specific utility of the biomaterials:

## Doing Good: Efficacy

New biomaterials give us a wonderful opportunity to do good and improve the *status quo*. It might be possible to probe previously explored phenomena in a new way and reveal hidden new phenomena due to deployment of novel functional biomaterials (McIntyre et al., 2004). We might *accelerate* or *enable* healing that was not previously possible when we combine interactive biomaterials (Hubbell, 1995) and potent inducers (Wozney et al., 1988). Such beneficial outcomes might be possible because biomaterials, when developed in the right way, have the potential to domesticate the nature, that is, to prevent its detrimental aspects from taking over an injury while enabling native processes to undertake the healing activity. This should excite the practitioners in the field, while energizing the scientist, the engineer, and the clinician to amalgamate their know-how and come up with creative solutions. The collective wisdom in the field can foster us to come up with creative solutions to our problems. These solutions may come from simply borrowing ideas from other fields and making use of biomaterials in a way that was not previously imagined, such as the idea of “printing” biological matter (Giordano et al., 1996). Alternatively, we might have to rely on novel chemical approaches to come up with totally synthetic or semi-synthetic materials with unique features and properties (Lee et al., 2005; Verheyen et al., 2011). Endogenous chemicals and architectures can guide new biomaterials for previously unforeseen applications. While nature can guide us toward uniquely beneficial features, we could come up with features un-matched in nature. We might have to explore physical means of creating our functional materials from “self” or “forced” assemblies (Chen et al., 2014), or rely on biological systems to obtain synthetic biomaterials that could not be envisioned before (Khalil and Collins, 2010; Leszczak et al., 2014). For the latter, one can envision cellular factories genetically engineered to synthesize, assemble, and deposit complex materials ready for tissue regeneration. Advanced computational techniques will need to be employed to better understand a regeneration process induced by biomaterials (Mousavi et al., 2013) or gene delivery systems (Meneksedag-Erol et al., 2014), with the intention of improving the design of biomaterials. Temporal and spatial information could be obtained with computational tools that are not readily available by current experimental approaches, such as the molecular basis for effective organization of self-assembling building blocks. Doing good, however, needs to be undertaken expeditiously and in the context of financial and ethical pressures facing our society. We do not have the luxury of working at a leisurely pace while the society is expecting cost-effective solutions to partially met or unmet clinical problems. This can be achieved by relying on innovative biomaterials that are functional at molecular level, fabricated devices that are exquisitely designed and engineered at the nano–micro–meso scale and assessed comprehensively in relevant biological systems. Let there be no doubt that we can ask our biomaterials to jump higher and run faster while saving lives and reducing our financial burden.

## Doing No-Harm: Safety

One cannot overestimate the unpredictable nature of living systems and their ability to adopt and respond in the face of an intrusion. Nature has a way of reminding us of the limitations of our good intentions, whether we attempt to maintain our circulation with artificial devices (clotting problem; Nosé et al., 1967), or deliver a gene to support a failing physiology (genotoxicity; Hacein-Bey-Abina et al., 2003). We need to minimize or preferably eliminate the adverse impact of biomaterials on biological systems. This calls for using naturally existing materials that can participate in endogenous processes of metabolism and elimination, or rely on building blocks that are harmless to biological systems. We need to keep a focus on different scales of biological systems when attempting to do no-harm; the molecular scale where perturbations of native biomolecular structures need to be avoided, the cellular scale where adverse effects manifest themselves as a result of physical alterations in sub-cellular structures, tissue/organ scales where the special anatomical structures, such as the junctional alignment of cells or tubular arrangement of blood vessels, should not be compromised. These considerations are amplified especially when we create designer topographies or nano-structured materials (Shvedova and Kagan, 2010, Koegler et al., 2012). The best way to ensure “no-harm” is to rely on degradable biomaterials that leave no traces of the biomaterial behind after the biomaterial completes its task in the body (Bianco et al., 2011; Dürig et al., 2011), and with degradation products that are either excreted directly or can enter into natural metabolic pathways. One cannot take a narrow approach to safety; a complementary analysis of body fluids, immune, and other surveillance systems have to be vigilantly probed to ensure biomaterial compliance with the biological system in the short term. Given the possibility of altering the expression of a wide range of genes whenever a therapeutic agent is introduced into the body, long-term assessments for genotoxicity of the biomaterials will be needed. At the end of the day, we should be on guard to expect a surprise from the nature, but also be confident that progress will be made by overcoming the presented obstacles. Preclinical models, designed for the intended application [after all, “biocompatibility” is inherently linked to an application (Williams, 2008)] and characterized thoroughly, are our best tools to pursue no-harm before the biomaterials find their way to clinical utility (Byrom et al., 2010; Muschler et al., 2010; Fitzgerald et al., 2011).

## Future of Biomaterials

While we focus on doing good and ensuring no-harm, it is clear to me that our challenges are closely tied to the problems that we are attempting to solve. This makes it difficult to focus on a few overarching or grand challenges, which thinly disperses our collective wisdom throughout the field. However, I believe that it is still possible to identify several fundamental challenges that are critical to the biomaterials field and can lead to quantum leaps.

### Emulating nature for new materials with unique functionalities

Nature has given us materials that display the highest strength, strongest adhesion, greatest flexibility, error-free duplication, induced degradation, and unique on-demand features, to name a few qualities. While we are learning to mimic nature to reach its level of sophistication with synthetic biomaterials, we might even do better than what we are emulating when we apply our ingenuity and tools fully. Super-adhesive biomaterials (Brubaker and Messersmith, 2012), for example, might one day seal vascular defects at a fraction of the time that is possible today after simply injecting the biomaterials in the circulatory system. Artificial cells (Forster and Church, 2006), whose development was put aside in favor of native cells, might be deployed in the fight against cancer in the future. An in-depth understanding of natural materials, especially their operational principles, is paramount in this endeavor.

### Controlling the organization of matter

Desired outcomes from biomaterials are closely related to the organization of their building blocks. For some applications, organization at atomic scale is paramount, while other applications may call for organization at a specific region of the nano- or micro-scale. By assembly of right building blocks of a biomaterial at the appropriate scale, and then employing an engineered and controllable fabrication process for a functional device, we should be able to fulfill the promise of the biomaterials more fully. Creating nanoparticles with the ability to self-propel and move toward, a chemo-attractant might be feasible with structures that are assembled from functionally organized domains. Given the inherent difficulties of fabricating structurally controlled materials and devices for the first time, computational approaches could be a good start for exploring hypothetical scenarios for the organized matter.

### Designing the interface between biomaterials and biological systems to modulate responses

It is well recognized that biomaterial surfaces are distinctly different from their bulk features, be it their chemical composition or physical properties. We should deliberately control the surface features of biomaterials since all biological systems first get exposed and subsequently respond to this interface. For example, it may be possible to create sensors that function endlessly due to “stealth” surface features that make the body ignore their presence while they sense the immediate environment. We might have to induce “tolerance” to biomaterials for a stable, non-reactive interface, reminiscent of tissue transplantation (Bishop et al., 2011). For reactive interfaces intended to interact with their surroundings, the challenge of presenting biological cues in a sea of adverse host factors (proteolytic enzymes, protein adsorption, extracellular matrix deposition, etc.) remains to be addressed. Will it be possible to design biomaterials in such a way to target the right cellular process for an amplified/stable response, while the body is attempting to isolate or neutralize the foreign entity?

### Relying on inherent physiological mechanisms

We routinely relied on biostable materials for permanent solutions at a time when little was known about the inherent capacity of our bodies to respond to undesired circumstances. With rapidly accumulating information on molecular and cellular basis of pathophysiology and regeneration, time is ripe to rely on our inherent capabilities for healing, and biomaterials can be designed to take advantage of innate processes to repair failing tissues and organs. While this approach is beginning to fulfill its promise in certain cases, such as the bone and skin, its real return will be in the repair of vital organs that evaded regenerative healing, such as heart and brain. We will need to employ our entire arsenal in this regard to attract the right cells, control the fate of invading cells, be it morphogenetic differentiation, senescence, or apoptosis, while the host response to our intervention supports the intended outcome. Controlling the mechanical features of functional devices will be equally important in this endeavor due to their impact on cellular fate (Engler et al., 2006). How mechano-regulation can be implemented at a wound site, and not just under culture conditions, will be an important goal to achieve. Biomaterial scaffolds designed in the right way will play a significant role in this endeavor.

### Improving over “gold” standard

Our biomaterials, once fabricated into functional devices, should do better than the accepted practice when it comes to efficacy, side-effects, and costs; the expectation is to improve in all three aspects. Innovative fabrication technologies capable of handling multimodal biomaterials and other biological materials, such as regulatory molecules and cells, will be required to achieve this goal. Simple-to-produce DNA delivery systems functioning better than viruses, “off-the-shelf” kidneys for transplantation or artificial cells secreting insulin on-demand, produced by large-scale cost-effective manufacturing processes, might 1 day find their way to medical practice. Of course, strategies to prevent opportunistic infections are expected to be an integral part of each intervention. Disruptive discoveries will play a key role in setting up new gold standards. Knowing that induced pluripotent cells (Takahashi and Yamanaka, 2006) can one day shake up the foundation of tissue engineered devices, and microRNA can add a new dimension into regenerative medicine (Lee et al., 1993), we should not shy away from unsettling ideas and seek opportunities to actively incorporate such ideas into our practice. The best way to ensure improved gold standards is to engage clinicians in biomaterials development and overcome the challenges of cooperating across disciplines whose foci are varied.

We expect the studies published in the specialty section of *Biomaterials*, under *Frontiers in Bioengineering and Biotechnology* and *Materials*, to contribute to solving our current challenges, while defining new challenges for the future.

## Conflict of Interest Statement

The author declares that the research was conducted in the absence of any commercial or financial relationships that could be construed as a potential conflict of interest.
